# *Cytauxzoon paradoxurus* n. sp., a novel *Cytauxzoon* species identified in common palm civets in Singapore

**DOI:** 10.1186/s13071-025-06820-0

**Published:** 2025-05-15

**Authors:** Stacy Q. Y. Chong, Darren Yeo, Alaine V. V. Arceo, Jasmine L. Y. Ong, Christine H. E. Lee, Rachael J. Y. Yeak, Alvin S. Z. Wee, Petrina Y. Z. Teo, Moses K. J. Tay, Amy H. J. Chan, Charlene Judith Fernandez, Renhui Xie, Anna M. S. Wong, Choon Beng How, Siow Foong Chang

**Affiliations:** Animal and Veterinary Service, National Parks Board (NParks), 1 Cluny Road, Singapore Botanic Gardens, Singapore, 259569 Singapore

**Keywords:** Civets, Wildlife, *Cytauxzoon*, Piroplasm, Tick-borne pathogen, Southeast Asia

## Abstract

**Background:**

The common palm civet (*Paradoxurus musangus*) is a species native to Southeast Asia. Highly adapted to urbanised environments, these civets can often be found in proximity to humans and companion animals, raising the concern of pathogen transmission at the human-wildlife and wildlife-domestic animal interface. Whilst there have been reports of various bacteria and viruses detected in civets, little is known about the protozoa that they may harbour. In this study, we screened the common palm civets in Singapore for tick-borne protozoan parasites known as piroplasms.

**Methods:**

Over a 2-year period, blood samples were opportunistically collected from 135 wild common palm civets following a physical examination. The sex and weight of each civet were recorded, and any ectoparasites detected were identified through DNA barcoding. DNA extracts of blood samples were screened using a PCR assay targeting the 18S rRNA gene of piroplasmids.

**Results:**

A novel *Cytauxzoon* species was detected in 29 civets (21.5%), and a statistically significant association was found between infection and the civet’s weight. Two cat flea (*Ctenocephalides felis*) specimens were discovered on two sampled civets; however, *Cytauxzoon* DNA was not detected in either the flea or the sampled civet. Phylogenetic analysis of the *Cytauxzoon 18S* rRNA gene sequences from 29 civets revealed that this piroplasmid is most closely related to a *Cytauxzoon* sp. detected in meerkats in South Africa but molecularly distinct from the six currently described *Cytauxzoon* species.

**Conclusions:**

This detection documents the first molecular confirmation of *Cytauxzoon* sp. infection in Southeast Asia and the first report of *Cytauxzoon* sp. in a viverrid host. Further studies are required to determine the vector involved in the transmission of this novel *Cytauxzoon* species, as no ticks were found on the sampled civets. The discovery of *Cytauxzoon paradoxurus* n. sp. highlights the importance of expanded biosurveillance to better understand the diversity of piroplasms harboured by wildlife in the region and its potential for cross-species transmission.

**Graphical Abstract:**

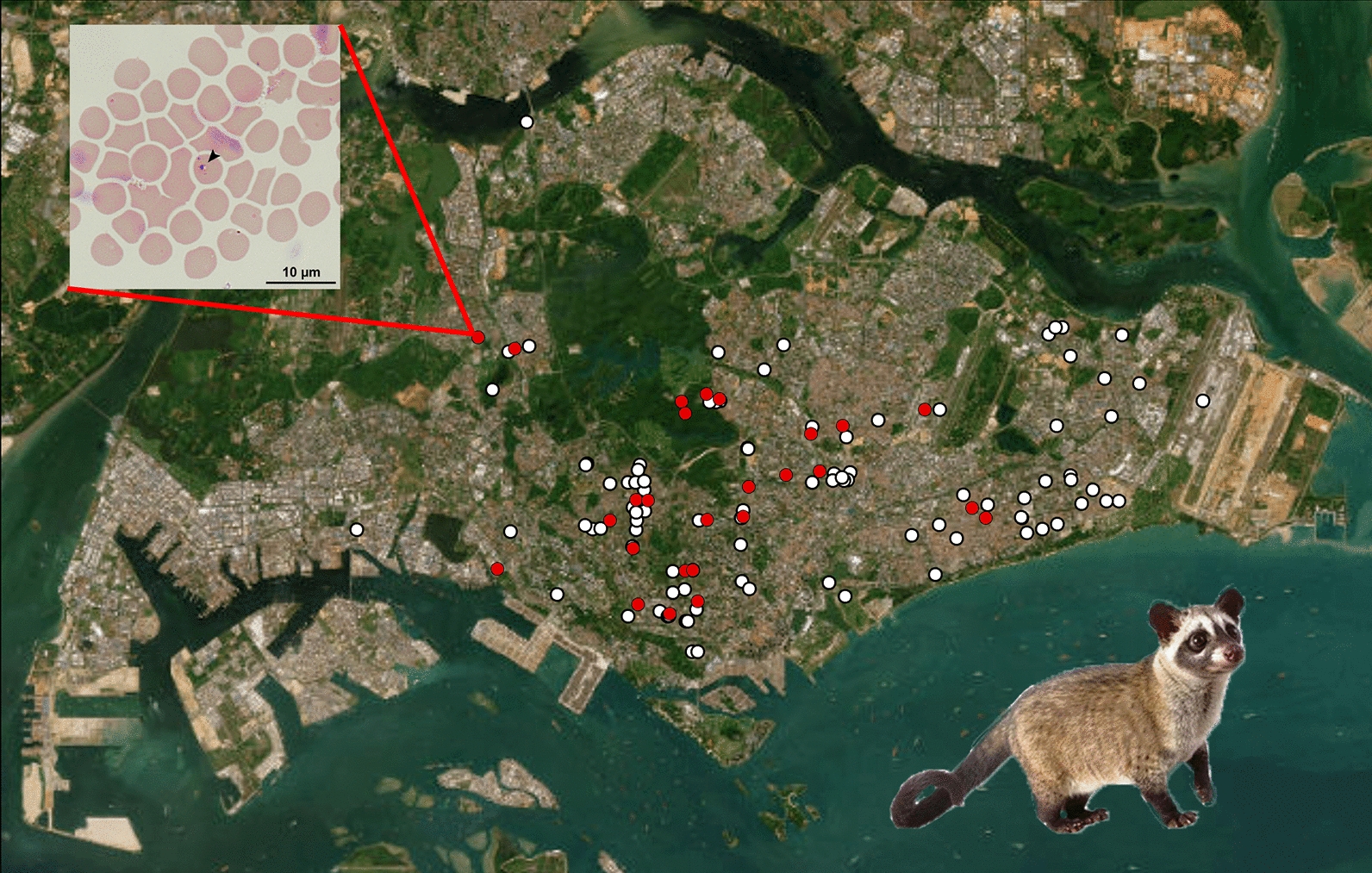

**Supplementary Information:**

The online version contains supplementary material available at 10.1186/s13071-025-06820-0.

## Background

Members of the Piroplasmida order comprise a diverse group of haemoprotozoan parasites, including those from the genera *Babesia*, *Cytauxzoon* and *Theileria*. Piroplasms, which are transmitted by tick vectors, are one of the most prevalent blood parasites in domestic and wild mammals globally [1]. Wildlife hosts are known to play a critical role as reservoirs of these parasites. As such, with habitat loss and fragmentation driving their growing proximity to humans, it is imperative to study the diversity of piroplasms harboured by wildlife and their potential for zoonotic transmission.

Civets (Viverridae) are one such forest-dwelling species that have readily adapted to rapid urbanisation in Asia. These nocturnal, omnivorous mammals have been reported to be highly susceptible to a wide range of bacteria, viruses and protozoa, some of which are capable of infecting humans [2]. Notably, masked palm civets were implicated in the emergence of severe acute respiratory syndrome coronavirus (SARS-CoV) in China, with molecular evidence suggesting they may have bridged the transmission from its natural host to humans [3]. Pathogens that can cause potentially fatal diseases in dogs or cats, such as canine and feline parvoviruses [4, 5], canine distemper virus [6] and *Leptospira interrogans* [7], have also been detected in different civet species worldwide. Piroplasms, however, have not been well studied in the Viverridae, with only a single report from 1968 of a *Babesia*-like piroplasm observed in blood films from common palm civets in Vietnam [8].

Common palm civets are widely distributed in South and Southeast Asia, and following a taxonomic revision in 2015, a new species named *Paradoxurus musangus* was proposed to describe populations native to mainland Southeast Asia and parts of Indonesia [9]. In Singapore, *P. musangus* frequently lives in or near human habitation, particularly where there are fruit trees for sustenance or nesting spaces in roofs or ceilings [10]. This proximity to humans and their companion animals raises the concern of zoonotic spillover and cross-species transmission of pathogens. In this study, we survey the local wild population of common palm civets for piroplasms, identify and describe a new genetically distinct *Cytauxzoon* species, *Cytauxzoon paradoxurus* n. sp., and investigate its phylogenetic relationships.

## Methods

### Sampling

A total of 135 wild common palm civets across Singapore were opportunistically sampled in rescue and release operations carried out by the National Parks Board from 2022 to 2023. To facilitate low-stress handling, civets were sedated with an intramuscular injection of Zoletil^®^ (Virbac Laboratories, France) at 5 mg/kg or a combination of medetomidine (Sedator^®^, Dechra, UK) at 0.05 mg/kg and ketamine (Ceva Animal Health Pty Ltd, Australia) at 4 mg/kg before a physical examination was conducted. Their sex and weight were recorded, and any ectoparasites were collected and stored in 70% ethanol. Blood was sampled using jugular venipuncture with a 23G needle and stored in an EDTA tube. For each blood sample, two thin smears were prepared, air-dried, fixed with absolute methanol and then stained with either Diff-Quik or Giemsa.

### Molecular screening of blood samples

DNA was extracted from 200 µl of EDTA blood using the DNeasy^®^ Blood and Tissue kit (QIAGEN, Germany) as per the manufacturer’s instructions and then stored at − 20 °C pending further testing. All DNA extracts were screened using a piroplasmid-specific primer pair (BJ1/BN2) targeting an approximately 400–500 base pair (bp) partial region of the *18S* rRNA gene [11]. PCR was performed in a 25-µl reaction mixture containing 5 µl of DNA template, 1X Promega Green GoTaq^®^ Flexi Buffer, 0.1 mM of each dNTP, 2 mM of MgCl_2_, 350 nM of forward and reverse primers and 1 U of GoTaq DNA polymerase (Promega, USA). Thermal cycling conditions were set as follows on a ProFlex PCR System (Thermo Scientific, USA): initial denaturation at 95 °C for 2 min, followed by 35 cycles of activation at 95 °C for 30 s, annealing at 58 °C for 45 s and elongation at 72 °C for 45 s, ending with a final extension at 72 °C for 5 min. PCR products were visualized using a 1.5% agarose gel.

PCR-positive samples were subjected to a second PCR using the primer pair Piro18S_Frag1 F/Piro18S_Frag2R to amplify near full-length *18S* rRNA sequences. PCR cycling parameters were as described by Baneth et al. [12]. Amplicons were sent to a commercial company (Bio Basic Asia Pacific Pte Ltd, Singapore) for PCR clean-up and Sanger sequencing to obtain bidirectional sequences.

### Microscopic examination of blood smears

To visualise the morphology of piroplasms detected, blood smears from PCR-positive civets were examined at 1000 × magnification using an oil immersion lens with the Olympus BX53 microscope (Olympus, Japan). The piroplasms observed were then imaged at two separate facilities, using the Eclipse Ni-E microscope integrated with a DS-10 camera (Nikon, Japan) and the APX100 digital imaging system (Olympus, Japan) fitted with the ORCA-Fusion Digital CMOS camera (Hamamatsu Photonics, Japan).

### Data analyses

Sequences were processed and trimmed to remove priming sites and unreliable regions using Geneious Prime [13]. A BLAST search was performed against NCBI GenBank to obtain putative matches. *Cytauxzoon 18S* rRNA sequences were downloaded from GenBank (accessed on 3 February 2025) via its Taxonomy search (search term, “*Cytauxzoon*”) as well as additional “18S” and “small subunit” search terms. Sequences between 1000 and 2000 bp were retained for further analyses, while the host species and country of origin were recorded where available (Additional file 1: Table S1). Two outgroup sequences from *Theileria bicornis* (AF499604) and *Babesia caballi* (Z15104) were included. The sequences were aligned in MAFFT v7 [14] under default parameters except with the “adjust direction according to the first sequence” option selected. The alignment was screened in Aliview [15] to exclude non-homologous sequences.

ModelTest-NG [16] was used with the alignment and default parameters to determine which model was most suitable for tree reconstruction. A maximum likelihood (ML) tree was then generated from the aligned sequences in raxmlGUI 2.0 [17] with the GTR GAMMA model, invariant sites (+ I) and rapid bootstrapping with 1000 bootstrap replicates. The mammalian host topology with which the ML tree was depicted was obtained from Upham et al. [18]. The best ML tree was used for Poisson Tree Process (PTP) analysis [19] on the PTP web server (https://species.h-its.org/ptp, accessed on 20 February 2025) with default parameters and outgroups specified. The results for the ML iteration of PTP (mPTP) were reported in this study as this algorithm performs better than its Bayesian counterpart (bPTP) for fewer species [20]. Mean inter- and intra-specific pairwise distances (p-distances) between the molecular operational taxonomic units (mOTUs) designated by mPTP (*Cytauxzoon banethi*, *C. otrantorum* and *C. manul*) were analysed as separate species from *C. europaeus* [21], while *C. brasiliensis* was analysed as a separate species from *C. felis* [22]); they were calculated in MEGAX [23] using the same *18S* rRNA alignment utilized for tree reconstruction. In this analysis, uncorrected p-distances were used, including both transitions and transversions, as well as uniform rates, pairwise deletions for missing data and 1000 bootstrap replicates.

To investigate the potential factors associated with *Cytauxzoon* infection in Singapore’s common palm civets, a generalized linear model with binomial errors was constructed in R 4.3.2 [24] with *Cytauxzoon* detection as the response variable and weight, sex and sampling month as explanatory variables (Additional file 2: Dataset S1). The model was refined in a stepwise manner by removing the explanatory variable with the highest *P*-value and re-running the analysis until only explanatory variables with significant *P*-values (< 0.05) remained. Model AIC and analysis of deviance tests were used to determine whether the variable removal negatively impacted the model.

## Results

### Detection of a novel *Cytauxzoon* species in civet blood

Of the 135 screened common palm civets (79 male, 56 female), *Cytauxzoon* was detected in 29 samples (21.5%). No significant difference was observed in the proportion of infected civets sampled in 2022 (21.7%) and 2023 (21.3%). All sampled civets were confirmed to be *P. musangus* by comparing their cytochrome-b barcodes (representative sequence deposited in GenBank: PV402286) to references from Veron et al. [9]. A BLAST search of the *18S* rRNA gene sequences derived from the 29 infected civets revealed no identical species in GenBank, with the highest match identity at 96.53–97.73% to an unclassified *Cytauxzoon* sp. detected in meerkats in South Africa (GenBank accession number KM025200), and a 95.57–96.75% similarity to *Cytauxzoon felis* (GenBank accession nos. AF399930, GU903911).

**Fig. 1:**
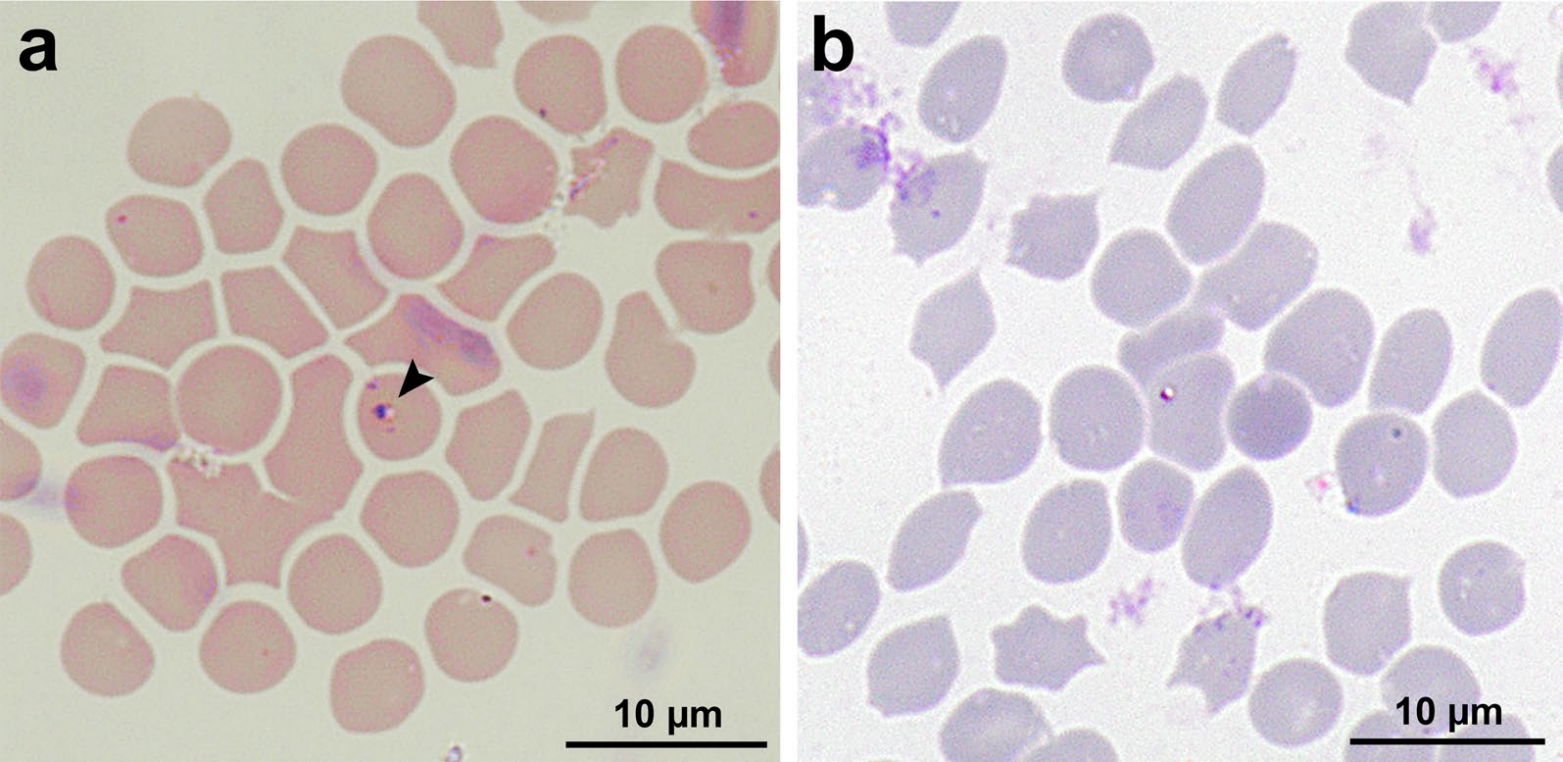
Erythrocytes infected with piroplasms observed in (**a**) DiffQuik and (**b**) Giemsa-stained thin blood smears from *Paradoxurus musangus* (magnification 1000 ×). The holotype is marked with an arrow. *Scale bars*: 10 μm

Single intra-erythrocytic inclusions measuring 1–2 µm, characterized by a distinct dark eccentric nucleus and pale to hyaline cytoplasm, were observed on Diff-Quik and Giemsa stains (Fig. 1). The estimated percentage of parasitaemia is < 1%, and no parasite stages were observed in monocytes.

**Fig. 2:**
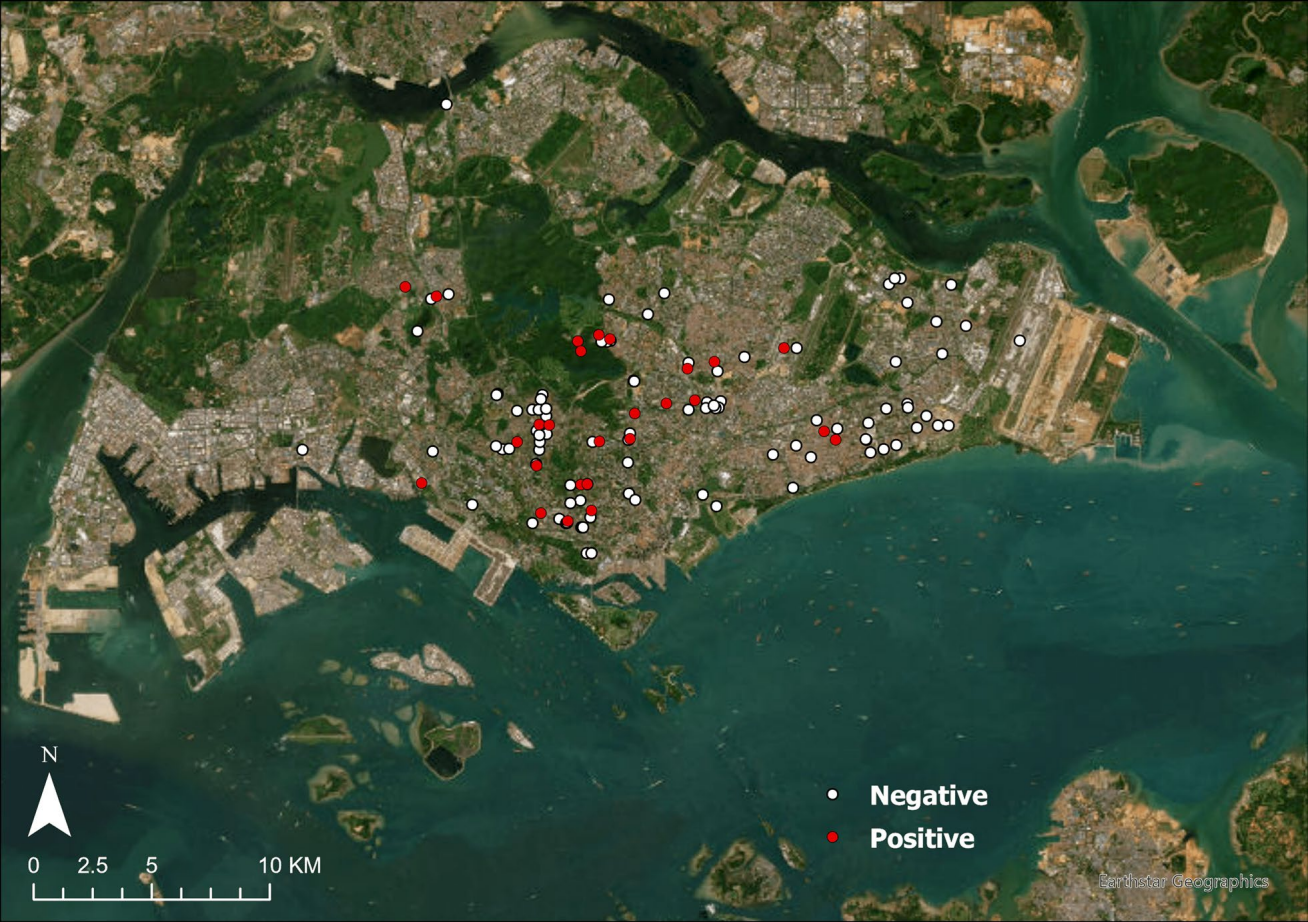
Location of civets sampled in this study. Positive *Cytauxzoon* detections are indicated in red, while negatives are in white. This map (basemap: World Imagery) was created with ArcGIS Pro version 3.4.0 (ESRI, Redlands, CA, USA)

Most of the sampled civets were from the central, southern and eastern regions of Singapore. *Cytauxzoon* was detected in civets across these three regions (Fig. 2), apart from the easternmost part of mainland Singapore. No statistically significant association was found between *Cytauxzoon* infection and the civet’s sex or sampling month. The final linear model with the lowest AIC had only weight as a highly significant explanatory variable (*P* < 0.001) at a weight coefficient of 1.4902. After applying the logit link function, the model predicts that the likelihood of *Cytauxzoon* infection increases by 4.4 per kg increase in weight (Additional file 3: Fig. S1).

Fleas were observed on two uninfected civets, and a single specimen was collected from each for molecular identification and screening. DNA barcoding using a fragment of the *COI* gene confirmed both specimens were the cat flea (*Ctenocephalides felis*; deposited in GenBank as PV403639 and PV403640). However, piroplasmid DNA was not detected in the fleas.

### Phylogenetics and species delimitation

**Fig. 3:**
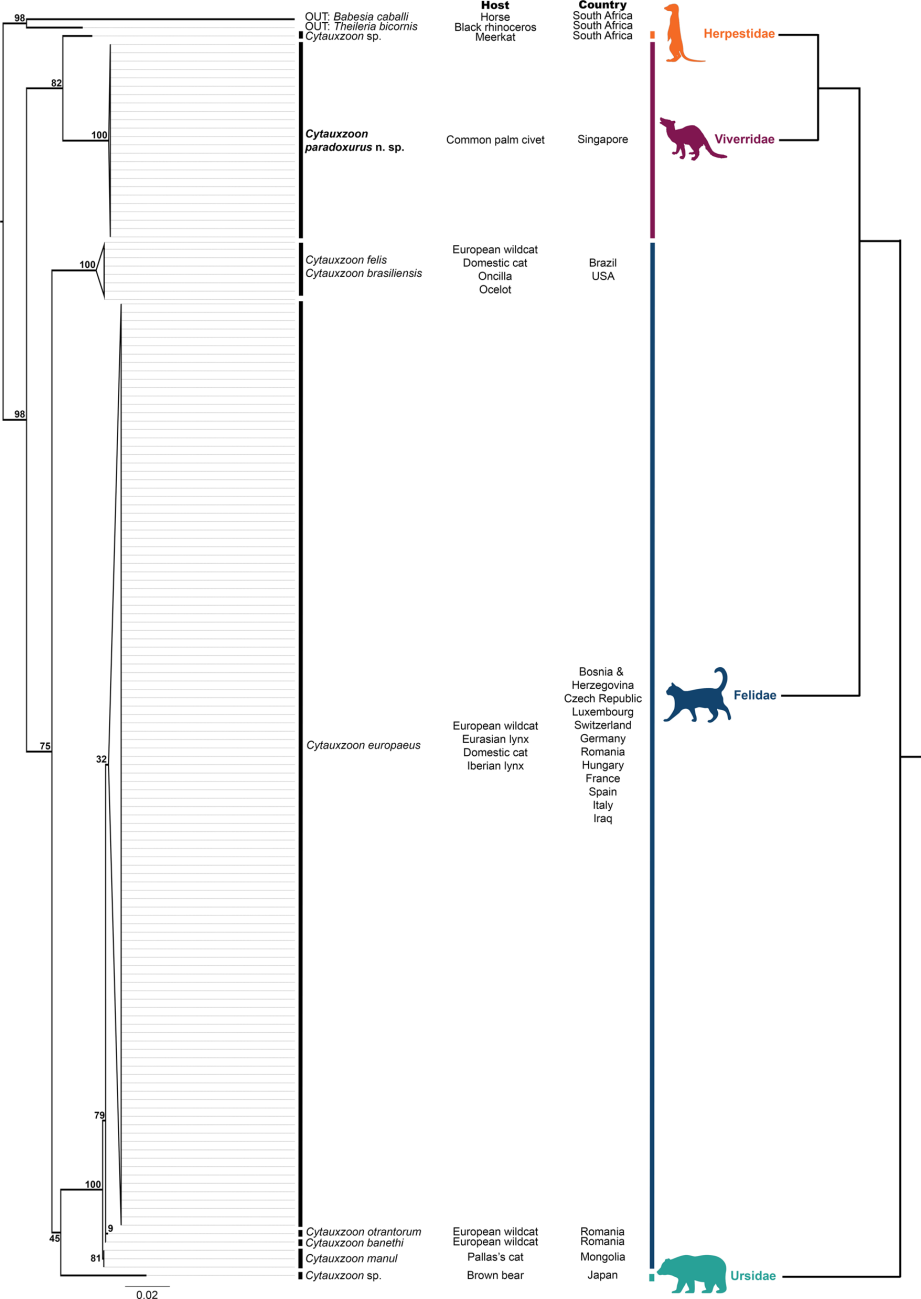
ML tree of *18S* rRNA sequences from *Cytauxzoon* species, including those downloaded from NCBI GenBank and this study (*left*), coupled with the tree topology of their mammalian hosts (*right*). Host species and country of origin for each *Cytauxzoon* species are listed in the centre

*Cytauxzoon 18S* rRNA gene sequences of > 1000 bp in length were obtained from 24 of the 29 infected civets and deposited in the GenBank database under accession numbers PV437184–PV437207. ModelTest-NG recommended the use of the GTR GAMMA model with invariant sites (GTR + G4 + I), which was then used for ML tree reconstruction [16]. The tree generated from *Cytauxzoon 18S* rRNA gene sequences (> 1000 bp) consisted of two outgroups and 149 terminals (24 from this study; 125 from GenBank) (Fig. 3). The clades corresponding to *Cytauxzoon felis* (Americas, with *C. brasiliensis* nested within), *C. manul*, *C. europaeus* and *Cytauxzoon* sp. (*Paradoxurus* host), are monophyletic and have been collapsed, while the other species have only one representative. Bootstrap support for most of the basal nodes is reasonably strong (> 75%), with the exception of the clade containing *Cytauxzoon felis* (Americas) and *Cytauxzoon* sp. (*Ursus* host) (43) and *C. europaeus.* (35). The topology of the *Cytauxzoon* species compared to that of their mammalian hosts is similar, except for the placement of *Cytauxzoon* sp. (*Ursus* host).

**Fig. 4:**
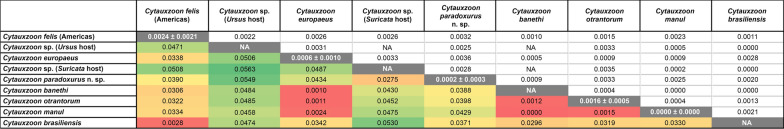
Inter- and intraspecific distances between various *Cytauxzoon* species. Interspecific p-distances are coloured in a heatmap from lowest (red) to highest (green) in the bottom left, while their standard deviations are in the top right. Intraspecific p-distances and standard deviations are indicated in the grey cells

The mPTP analysis yielded five *Cytauxzoon* mOTUs: (1) a species complex consisting of *Cytauxzoon felis* (Americas) and *C. brasiliensis*, (2) *Cytauxzoon* sp. (*Ursus* host), (3) *Cytauxzoon* sp. (*Suricata* host), (4) *Cytauxzoon* sp. (*Paradoxurus* host) and (5) a species complex consisting of *C. europaeus, C. banethi, C. otrantorum* and *C. manul* (Additional file 4: Fig. S2). The bPTP analysis yielded 23 species instead (Additional file 5: Fig. S3). The mean *18S* intraspecific p-distances (Fig. 4) ranged from 0 to 0.0024, with the smallest distances being within *C. manul* (*M* = 0, SD = 0), while the largest was within *C. felis* (Americas) (*M* = 0.0024, SD = 0.0021). The mean interspecific distances range from 0 to 0.0563, with the smallest distance being between *C. manul* and *C. banethi* (*M* = 0, SD = 0), while the largest distance was between *Cytauxzoon* sp. (*Suricata* host) and *Cytauxzoon* sp. (*Ursus* host) (0.0563). Of note, the smallest mean interspecific distance between *Cytauxzoon* sp. (*Paradoxurus* host) and its sister species *Cytauxzoon* sp. (*Suricata* host) is 0.0275 (SD = 0.0028). This is much larger than the highest mean interspecific distance between *C. europaeus*, *C. banethi*, *C. otrantorum* and *C. manul* (*M* = 0.0024, SD = 0.0009).

**Taxonomic review and species description**

**Family Theileriidae du Toit, 1918**

**Genus Cytauxzoon Neitz and Thomas, 1948**

**Cytauxzoon paradoxurus n. sp.**

***Type-host***: Common palm civet, *Paradoxurus musangus* Veron, 2014 (Carnivora, Viverridae, Paradoxurinae).

***Type-locality***: Singapore (1.301589, 103.822374).

***Type-material***: Genomic DNA extract from the blood of *P. musangus* (BIOS32) and stained thin blood smear (BIOS943, Fig. 1a) were deposited at Animal and Plant Health Centre, Singapore.

***Vector***: currently unknown.

***Representative DNA sequence: 18S*** rRNA amplified from the type material, deposited in GenBank under accession no. ON606000.

***ZooBank registration***: To comply with the regulations set out in article 8.5 of the amended 2012 version of the International Code of Zoological Nomenclature (ICZN), details of the new species have been submitted to ZooBank. The Life Science Identifier (LSID) of the article is urn:lsid:zoobank.org:pub: 9BCAD1 F3-8804-44 FE-BAB4-BC67D8 A8 F628. The LSID for the new name *Cytauxzoon paradoxurus* is urn:lsid:zoobank.org:act: 50983275-4828-41 CD-8 A7 A-EF4997EC344 C.

***Etymology***: The new species is named after the genus of the host species in which it was first discovered, *P. musangus*.

## Discussion

This study documents the first detection of a *Cytauxzoon* species in common palm civets from the Viverridae family. Prior to this, only two instances of *Cytauxzoon* infections have been reported outside the Felidae—one in the Herpestidae in meerkats (*Suricata suricatta)* in South Africa [25] and the other in the Ursidae in Hokkaido brown bears (*Ursus arctos yesoensis*) and Japanese black bears (*Ursus thibetanus japonicus*) in Japan [26]. This survey demonstrated that a *Cytauxzoon* species was present in 21.5% of common palm civets from Singapore. Compared to *Cytauxzoon* detections in other non-felid hosts, this is relatively lower than the reported incidence of 57% in meerkats and 91.8–95.2% in bears.

This detection also serves as the first molecular confirmation of *Cytauxzoon* sp. infection in Southeast Asia. Although *Cytauxzoon* was identified in Mongolian-caught Pallas'cats (*Otocolobus manul*) as early as 2005 [27], with subsequent reports in Japan [28], India [29], Iran [30] and most recently China [31] and Korea [32]; its prevalence in domestic and wild animal populations in Asia remains vastly understudied to date, particularly in Southeast Asian. In Singapore, the widespread distribution of infected civets and the stable incidence rates over the 2-year study period seem to suggest that *C. paradoxurus* n. sp. is enzootic in local populations of common palm civets. However, molecular surveys of felids, which are susceptible to *C. felis*, have not been performed, and it is not known if other *Cytauxzoon* species are also prevalent in Singapore.

As species diagnosis of piroplasmids is often not possible through microscopic examination because of shared morphological features between different genera, molecular methods are crucial to reliably distinguish between different species as well as to delineate novel species [33]. The genotypic data from this study support the status of this *Cytauxzoon* in common palm civets as a novel species, distinct from the five species currently described. The *18S* rRNA gene is a conserved marker widely used for piroplasmid detection and identification. The mean *18S* interspecific distances (Fig. 4) between *C. paradoxurus* n. sp. and its next closest known species [*Cytauxzoon* sp. (*Suricata* host)] is 2.75% (SD = 0.28), which is much larger than the mean interspecific *18S* distances between formally named and described *Cytauxzoon* species, *C. europaeus*, *C. banethi*, *C. otrantorum* and *C. manul* (0.00–0.24%) [21]. Its mean intraspecific distance is also extremely low (0.02%, SD = 0.03%), indicating a clear barcoding gap between inter- and intraspecific distances [34]. This is further supported by our mPTP results (Additional file 3: Fig. S3), which classify *C. paradoxurus* n. sp. as a distinct species based on monophyly and substitution rates [35]. Of note, mPTP classifies the four aforementioned named *Cytauxzoon* species as a single species. We disregarded the bPTP classification of 23 species as the algorithm is known to over-split species [20]. We believe that by using these widely used genetic species delimitation approaches, our results satisfy the burden of proof to characterize *C. paradoxurus* n. sp. as a new species under the phylogenetic species concept sensu Mishler and Theriot [36].

The species status of *C. paradoxurus* n. sp. is reinforced from an evolutionary perspective by thus far being only detected in the common palm civet. While this could be due to overall poor piroplasmid screening efforts of wildlife in the region, it is notable that the sister clade to *C. paradoxurus* n. sp. in our analysis is *Cytauxzoon* sp. (*Suricata* host), which mirrors the close relationship between the Viverridae and Herpestidae clades in the mammalian tree of life [18]. This supports the hypothesis that *C. paradoxurus* n. sp. could have co-evolved with its mammalian host, diverging when the last common ancestor of Viverridae and Herpestidae speciated (est. 20.2 Mya). However, this is confounded by the placement of *Cytauxzoon* sp. (*Ursus* host) in our tree, which is derived from the clade of *Cytauxzoon* species that infect felids. Its placement is in contrast to the mammalian tree where the Ursidae branches off from the common ancestor of Felidae, Viverridae and Herpestidae [18]. This could be due to a more recent host shift from felids to ursids, but this requires further investigation as the support for that node is too weak to draw any concrete conclusions.

The hypothesis that *P. musangus* is the natural host of *C. paradoxurus* n. sp. is further supported by the observations that infected civets did not appear to exhibit any overt clinical signs upon physical examination as well as the absence of schizonts upon microscopic examination of blood smears. Although additional laboratory tests such as haematology and biochemistry analyses are required to study the pathogenicity of *C. paradoxurus* n. sp. in civets, the lack of apparent clinical disease is consistent with *C. felis* infections in bobcats (*Lynx rufus*)—the natural host and primary sylvatic reservoir of *C. felis* in North America [37, 38]. In bobcats, *C. felis* has been shown to undergo a shortened schizogenous phase of asexual replication within macrophages, following which the parasite persists within erythrocytes without causing clinical disease [39]. This chronic, low parasitaemia in its natural host is generally thought to be an adaption of piroplasmids that allows the survival of its reservoir hosts whilst also extending the period in which a tick may acquire the parasite during feeding [40].

While the natural host may be non-clinical, it is important to note that the introduction of a parasite into a maladapted host species with an inadequate immune response can result in clinical manifestation of disease. In domestic cats, *C. felis* infection often results in acute, life-threatening disease when schizont-laden macrophages occlude blood vessels, causing multi-organ failure [41]. Despite advances in treatment methods, fatalities are still reported in at least 40% of cases [42]. Thus, enhanced biosurveillance is necessary to monitor the risk of cross-species transmission of *C. paradoxurus*, particularly at the wildlife-domestic animal interface.

The importance of biosurveillance is also highlighted by the presence of cat fleas (*Ct. felis*) on common palm civets. These cosmopolitan, haematophagous ectoparasites have previously been documented on owned dogs and cats in Singapore [43], but its observation on civets represents a novel host-parasite interaction and a potential parasite-sharing network between its primary hosts of cats and dogs and a wildlife species. This is not unexpected, as modelling of global datasets by Clark et al. [44] predicted that *Ct. felis* can infest an extensive range of host species across different mammalian families and revealed that wildlife species that use anthropogenic habitats are at a dramatically higher risk of infestation by cat fleas. Although *Ct. felis* is a known vector of numerous bacterial pathogens, thus far, no evidence has suggested fleas can transmit piroplasmids, and further studies are required to determine whether cat fleas play a role in the transmission of *C. paradoxurus* n. sp.

Like other piroplasmids, *Cytauxzoon* is known to be primarily transmitted by ticks. As tick infestation was not observed on any of the civets in our study, the vector involved in the transmission of *C. paradoxurus* n. sp. remains undetermined. Experimental studies have shown that the *Dermacentor variabilis* [45] and *Amblyomma americanum* [46] are competent vectors of *C. felis*, but their distribution is confined to the geographical region of North America, and little is known about the probable vectors of *Cytauxzoon* spp. in other continents. In addition, very few studies have examined the ectoparasites of common palm civets. Of note, a survey of a forest reserve in northeast Thailand discovered multiple tick species from the *Haemaphysalis*, *Ixodes* and *Amblyomma* genera, infesting seven civets at a mean intensity of 3.1 ticks [47]. Whilst these genera of ticks have also been reported in Singapore [48], the urban spaces inhabited by civets in our study are likely less favourable to tick survival compared to forested ones, which may explain the absence of ticks observed. Moreover, common palm civets engage in self-grooming and allogrooming behaviours that effectively allow the removal of ticks [49, 50]. More data on the distribution of tick species across various land-use gradients in Singapore may help provide more clues about the possible vector involved in the transmission of *C. paradoxurus* n. sp.

Vector-independent pathways may also be an alternative route of transmission for some piroplasms. However, while there is evidence of vertical transmission of certain *Theileria* and *Babesia* spp., there have been no confirmed reports of such for *Cytauxzoon* [51]. This is supported by our results, which revealed that civets of a higher weight were significantly more likely to be infected with *C. paradoxurus* n. sp. Since age and weight are highly correlated, this suggests that the parasite is less likely to be spread vertically from mother to offspring and more likely to be acquired by the host as it ages. Studies have shown that heavier rodents [52] and cattle [53] experienced higher tick burdens, resulting in an increased likelihood of acquiring tick-borne diseases, likely because of a larger body surface area for the vector to attach to and feed.

## Conclusions

We report the first detection of *Cytauxzoon* in Southeast Asia and describe a novel *Cytauxzoon* species, *C. paradoxurus* n. sp., that infects *P. musangus* in Singapore. Although no zoonotic cases of *Cytauxzoon* infection have been reported, expanded biosurveillance is crucial to understanding the diversity and prevalence of *Cytauxzoon* spp. in animal populations in the region and to identifying any emerging threats and minimizing the risk of spillover at the wildlife-domestic animal interface, especially given the increasing overlap of habitats shared by pets and wildlife.

## Supplementary Information

Additional file 1. Table S1. List of *Cytauxzoon *sequences downloaded from NCBI GenBank for phylogenetic analysis.

Additional file 2. Dataset S1. Weight, sex and month of sampling of *Cytauxzoon paradoxurus *n. sp.-infected and non-infected civets.

Additional file 3. Relationship between civet weight and *Cytauxzoon paradoxurus* n. sp. detectionin this study.

Additional file 4. Fig. S2. mPTP analysis

Additional file 5. Fig. S3. bPTP analysis

## Data Availability

All data generated or analysed during this study are included in this article and supplementary information files. The sequence data obtained from this study have been submitted to the NCBI GenBank database (accession nos. PV402286 for *Paradoxurus musangus*, PV437184–PV437207 for *Cytauxzoon paradoxurus* n. sp. and PV403639—PV403640 for *Ctenocephalides felis*).
