# Supplementary material for: Cytauxzoon paradoxurus n. sp., a novel Cytauxzoon species identified in common palm civets in Singapore
Source: Parasit Vectors. 2025 May 15;18:175. doi: 10.1186/s13071-025-06820-0 (PMC12079874; doi:10.1186/s13071-025-06820-0)
Supplement: Supplementary file 1 — Additional file 1. Table S1. List of Cytauxzoon sequences downloaded from NCBI GenBank for phylogenetic analysis. [file 13071_2025_6820_MOESM1_ESM.docx]

**Additional file 1: Table S1.** List of *Cytauxzoon* sequences downloaded from NCBI Genbank for phylogenetic analysis**.**

| ***Cytauxzoon* Species** | **Country** | **Host** | **Accession number** | **Remarks** |
| --- | --- | --- | --- | --- |
| *Theileria bicornis* | South Africa | *Diceros bicornis* | AF499604 | Outgroup |
| *Babesia caballi* | South Africa | *Equus caballus* | Z15104 | Outgroup |
| *Cytauxzoon banethi* | Romania | *Felis silvestris* | MT904029 |  |
| *Cytauxzoon europaeus* | Bosnia and Herzegovina | *Felis silvestris* | MT904025 |  |
| *Cytauxzoon europaeus* | Czech Republic | *Lynx lynx* | MT904036 |  |
| *Cytauxzoon europaeus* | Germany | *Felis silvestris* | MT904041 |  |
| *Cytauxzoon europaeus* | Germany | *Felis silvestris* | ON380467 |  |
| *Cytauxzoon europaeus* | Germany | *Felis silvestris* | ON380468 |  |
| *Cytauxzoon europaeus* | Germany | *Felis silvestris* | ON380470 |  |
| *Cytauxzoon europaeus* | Germany | *Felis silvestris* | ON380472 |  |
| *Cytauxzoon europaeus* | Germany | *Felis silvestris* | ON380477 |  |
| *Cytauxzoon europaeus* | Germany | *Felis silvestris* | ON380481 |  |
| *Cytauxzoon europaeus* | Germany | *Felis silvestris* | ON380469 |  |
| *Cytauxzoon europaeus* | Germany | *Felis silvestris* | ON380471 |  |
| *Cytauxzoon europaeus* | Germany | *Felis silvestris* | ON380476 |  |
| *Cytauxzoon europaeus* | Germany | *Felis silvestris* | ON380483 |  |
| *Cytauxzoon europaeus* | Germany | *Felis silvestris* | ON380484 |  |
| *Cytauxzoon europaeus* | Germany | *Felis silvestris* | ON380444 |  |
| *Cytauxzoon europaeus* | Germany | *Felis silvestris* | ON380447 |  |
| *Cytauxzoon europaeus* | Germany | *Felis silvestris* | ON380445 |  |
| *Cytauxzoon europaeus* | Germany | *Felis silvestris* | ON380461 |  |
| *Cytauxzoon europaeus* | Germany | *Felis silvestris* | ON380446 |  |
| *Cytauxzoon europaeus* | Germany | *Felis silvestris* | ON380453 |  |
| *Cytauxzoon europaeus* | Germany | *Felis silvestris* | ON380462 |  |
| *Cytauxzoon europaeus* | Germany | *Felis silvestris* | MT904040 |  |
| *Cytauxzoon europaeus* | Germany | *Felis silvestris* | ON380442 |  |
| *Cytauxzoon europaeus* | Germany | *Felis silvestris* | ON380448 |  |
| *Cytauxzoon europaeus* | Germany | *Felis silvestris* | ON380454 |  |
| *Cytauxzoon europaeus* | Germany | *Felis silvestris* | ON380459 |  |
| *Cytauxzoon europaeus* | Germany | *Felis silvestris* | ON380465 |  |
| *Cytauxzoon europaeus* | Germany | *Felis silvestris* | ON380455 |  |
| *Cytauxzoon europaeus* | Germany | *Felis silvestris* | ON380458 |  |
| *Cytauxzoon europaeus* | Germany | *Felis silvestris* | ON380450 |  |
| *Cytauxzoon europaeus* | Germany | *Felis silvestris* | MT904038 |  |
| *Cytauxzoon europaeus* | Germany | *Felis silvestris* | MT904039 |  |
| *Cytauxzoon europaeus* | Germany | *Felis silvestris* | ON380449 |  |
| *Cytauxzoon europaeus* | Germany | *Felis silvestris* | ON380475 |  |
| *Cytauxzoon europaeus* | Germany | *Felis silvestris* | ON380451 |  |
| *Cytauxzoon europaeus* | Germany | *Felis silvestris* | ON380452 |  |
| *Cytauxzoon europaeus* | Germany | *Felis silvestris* | ON380443 |  |
| *Cytauxzoon europaeus* | Germany | *Felis silvestris* | ON380480 |  |
| *Cytauxzoon europaeus* | Germany | *Felis silvestris* | ON380474 |  |
| *Cytauxzoon europaeus* | Germany | *Felis silvestris* | ON380478 |  |
| *Cytauxzoon europaeus* | Germany | *Felis silvestris* | ON380486 |  |
| *Cytauxzoon europaeus* | Germany | *Felis silvestris* | ON380485 |  |
| *Cytauxzoon europaeus* | Germany | *Felis silvestris* | ON380482 |  |
| *Cytauxzoon europaeus* | Hungary | *Felis silvestris* | OM256565 |  |
| *Cytauxzoon europaeus* | Italy | *Felis silvestris* | MT904034 |  |
| *Cytauxzoon europaeus* | Italy | *Felis silvestris* | MT904035 |  |
| *Cytauxzoon europaeus* | Italy | *Felis silvestris* | MT904026 |  |
| *Cytauxzoon europaeus* | Luxembourg | *Felis silvestris* | MT904044 |  |
| *Cytauxzoon europaeus* | Luxembourg | *Felis silvestris* | MT904043 |  |
| *Cytauxzoon europaeus* | Luxembourg | *Felis silvestris* | MT904042 |  |
| *Cytauxzoon europaeus* | Romania | *Felis silvestris* | MT904028 |  |
| *Cytauxzoon europaeus* | Romania | *Lynx lynx* | MT904027 |  |
| *Cytauxzoon felis* | Brazil | *Leopardus pardalis* | GU903911 |  |
| *Cytauxzoon felis* | Brazil | *Leopardus tigrinus* | DQ382277 |  |
| *Cytauxzoon felis* | Iraq | *Felis catus* | LC775709 |  |
| *Cytauxzoon felis* | Switzerland | *Felis catus* | KU306942 |  |
| *Cytauxzoon felis* | Switzerland | *Felis catus* | KU306944 |  |
| *Cytauxzoon felis* | Switzerland | *Felis catus* | KU306945 |  |
| *Cytauxzoon felis* | Switzerland | *Felis catus* | KU306940 |  |
| *Cytauxzoon felis* | Switzerland | *Felis catus* | KU306943 |  |
| *Cytauxzoon felis* | Switzerland | *Felis catus* | KU306946 |  |
| *Cytauxzoon felis* | Switzerland | *Felis catus* | KU306941 |  |
| *Cytauxzoon felis* | Switzerland | *Felis catus* | KU306948 |  |
| *Cytauxzoon felis* | Switzerland | *Felis catus* | KU306947 |  |
| *Cytauxzoon felis* | Unknown | Unknown | L19080 |  |
| *Cytauxzoon felis* | USA | *Felis catus* | AF399930 |  |
| *Cytauxzoon felis* | USA | *Felis catus* | AY679105 |  |
| *Cytauxzoon felis* | USA | *Felis silvestris* | MT904037 |  |
| *Cytauxzoon felis* | USA | *Felis silvestris* | MT904032 |  |
| *Cytauxzoon manul* | Mongolia | *Otocolobus manul* | AY485690 |  |
| *Cytauxzoon manul* | Mongolia | *Otocolobus manul* | AY485691 |  |
| *Cytauxzoon otrantorum* | Romania | *Felis silvestris* | MT904033 |  |
| *Cytauxzoon otrantorum* | Romania | *Felis silvestris* | MT904030 |  |
| *Cytauxzoon otrantorum* | Romania | *Felis silvestris* | MT904031 |  |
| *Cytauxzoon sp.* | France | *Felis catus* | EU622908 |  |
| *Cytauxzoon sp.* | France | *Felis silvestris* | MW727406 |  |
| *Cytauxzoon sp.* | France | *Felis silvestris* | MW727398 |  |
| *Cytauxzoon sp.* | France | *Felis silvestris* | MW727404 |  |
| *Cytauxzoon sp.* | France | *Felis silvestris* | MW727402 |  |
| *Cytauxzoon sp.* | Germany | *Felis catus* | MN629916 |  |
| *Cytauxzoon sp.* | Italy | *Felis catus* | OM004053 |  |
| *Cytauxzoon sp.* | Italy | *Felis catus* | OM004057 |  |
| *Cytauxzoon sp.* | Italy | *Felis catus* | OM004051 |  |
| *Cytauxzoon sp.* | Italy | *Felis catus* | OM004052 |  |
| *Cytauxzoon sp.* | Italy | *Felis catus* | OM004054 |  |
| *Cytauxzoon sp.* | Italy | *Felis catus* | OM004055 |  |
| *Cytauxzoon sp.* | Italy | *Felis catus* | OM004056 |  |
| *Cytauxzoon sp.* | Japan | *Ursus arctos* | AB480558 |  |
| *Cytauxzoon sp.* | Mongolia | *Otocolobus manul* | AF5314182 |  |
| *Cytauxzoon sp.* | Romania | *Felis silvestris* | KT361071 |  |
| *Cytauxzoon sp.* | Romania | *Felis silvestris* | KT361076 |  |
| *Cytauxzoon sp.* | Romania | *Felis silvestris* | KT361081 |  |
| *Cytauxzoon sp.* | Romania | *Felis silvestris* | KT361079 |  |
| *Cytauxzoon sp.* | Romania | *Felis silvestris* | KT361072 |  |
| *Cytauxzoon sp.* | Romania | *Felis silvestris* | KT361074 |  |
| *Cytauxzoon sp.* | Romania | *Lynx lynx* | KT361080 |  |
| *Cytauxzoon sp.* | South Africa | *Suricata suricatta* | KM025200 |  |
| *Cytauxzoon sp.* | Spain | *Felis catus* | AY309956 |  |
| *Cytauxzoon sp.* | Spain | *Lynx pardinus* | EF094468 |  |
| *Cytauxzoon sp.* | Spain | *Lynx pardinus* | EF094469 |  |
| *Cytauxzoon sp.* | Spain | *Lynx pardinus* | EF094470 |  |
| *Cytauxzoon sp.* | Spain | *Lynx pardinus* | AY496273 |  |
| *Cytauxzoon sp.* | Switzerland | *Felis catus* | MF503143 |  |
| *Cytauxzoon sp.* | Switzerland | *Felis catus* | MF503141 |  |
| *Cytauxzoon sp.* | Switzerland | *Felis catus* | MF503145 |  |
| *Cytauxzoon sp.* | Switzerland | *Felis catus* | MF503147 |  |
| *Cytauxzoon sp.* | Switzerland | *Felis catus* | MF503148 |  |
| *Cytauxzoon sp.* | Switzerland | *Felis catus* | MF503146 |  |
| *Cytauxzoon sp.* | Switzerland | *Felis catus* | MF503144 |  |
| *Cytauxzoon sp.* | Switzerland | *Felis catus* | MF503142 |  |
| *Cytauxzoon sp.* | Switzerland | *Felis catus* | MW727381 |  |
| *Cytauxzoon sp.* | Switzerland | *Felis catus* | MW727384 |  |
| *Cytauxzoon sp.* | Switzerland | *Felis catus* | MW727382 |  |
| *Cytauxzoon sp.* | Switzerland | *Felis catus* | MW727383 |  |
| *Cytauxzoon sp.* | Switzerland | *Felis catus* | MW727385 |  |
| *Cytauxzoon sp.* | Switzerland | *Felis catus* | MW727387 |  |
| *Cytauxzoon sp.* | Switzerland | *Felis catus* | MW727389 |  |
| *Cytauxzoon sp.* | Switzerland | *Felis catus* | MW727392 |  |
| *Cytauxzoon sp.* | Switzerland | *Felis catus* | MW727395 |  |
| *Cytauxzoon sp.* | Switzerland | *Felis catus* | MW727396 |  |
| *Cytauxzoon sp.* | Switzerland | *Felis catus* | MW727390 |  |
| *Cytauxzoon sp.* | Switzerland | *Felis catus* | MW727394 |  |
| *Cytauxzoon sp.* | Switzerland | *Felis catus* | MW727391 |  |
| *Cytauxzoon sp.* | Switzerland | *Felis catus* | MW727397 |  |
| *Cytauxzoon brasiliensis* | Brazil | *Leopardus tigrinus* | PP583821 |  |
