# Supplementary material for: Cytauxzoon paradoxurus n. sp., a novel Cytauxzoon species identified in common palm civets in Singapore
Source: Parasit Vectors. 2025 May 15;18:175. doi: 10.1186/s13071-025-06820-0 (PMC12079874; doi:10.1186/s13071-025-06820-0)

**Additional file 3: Fig. S1.** Relationship between civet weight and *Cytauxzoon paradoxurus* n. sp. detection (0 = negative, 1 = positive) in this study.


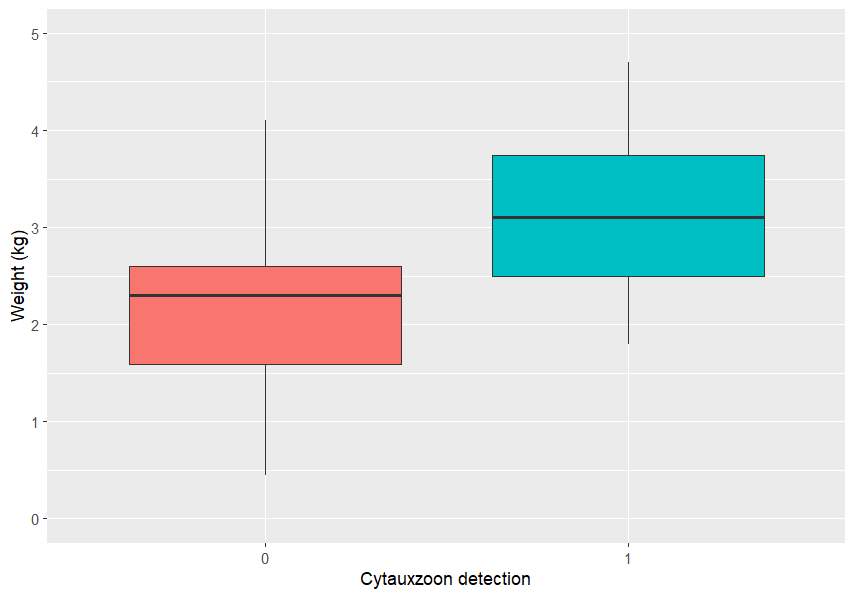

Supplement: Supplementary file 3 — Additional file 3. Relationship between civet weight and Cytauxzoon paradoxurus n. sp. detectionin this study. [file 13071_2025_6820_MOESM3_ESM.docx]
